# Weak population genetic structure in Eurasian spruce bark beetle over large regional scales in Sweden

**DOI:** 10.1002/ece3.9078

**Published:** 2022-07-06

**Authors:** Simon Jacobsen Ellerstrand, Shruti Choudhury, Kajsa Svensson, Martin N. Andersson, Carsten Kirkeby, Daniel Powell, Fredrik Schlyter, Anna Maria Jönsson, Mikkel Brydegaard, Bengt Hansson, Anna Runemark

**Affiliations:** ^1^ Department of Biology Lund University Lund Sweden; ^2^ Department of Forest Genetics and Plant Physiology, Umeå Plant Science Centre Swedish University of Agricultural Sciences Umeå Sweden; ^3^ Excellent Team for Mitigation, Faculty of Forestry & Wood Sciences Czech University of Life Sciences Prague Suchdol Czech Republic; ^4^ Animal Welfare and Disease Control Copenhagen University Frederiksberg C Denmark; ^5^ Global Change Ecology Research Group University of the Sunshine Coast Sippy Downs Queensland Australia; ^6^ Department of Plant Protection Biology Swedish University of Agricultural Sciences Lomma Sweden; ^7^ Department of Physical Geography and Ecosystem Science Lund University Lund Sweden; ^8^ Department of Physics Lund University Lund Sweden

**Keywords:** bark beetle, connectivity, gene flow, *Ips typographus*, migration, pest management, population structure

## Abstract

The Eurasian spruce bark beetle, *Ips typographus*, is a major pest, capable of killing spruce forests during large population outbreaks. Recorded dispersal distances of individual beetles are typically within hundreds of meters or a few kilometers. However, the connectivity between populations at larger distances and longer time spans and how this is affected by the habitat is less studied, despite its importance for understanding at which distances local outbreaks may spread. Previous population genetic studies in *I. typographus* typically used low resolution markers. Here, we use genome‐wide data to assess population structure and connectivity of *I. typographus* in Sweden. We used 152 individuals from 19 population samples, distributed over 830 km from Strömsund (63° 46′ 8″ N) in the north to Nyteboda (56° 8′ 50″ N) in the south, to capture processes at a large regional scale, and a transect sampling design adjacent to a recent outbreak to capture processes at a smaller scale (76 km). Using restriction site‐associated DNA sequencing (RADseq) markers capturing 1409–1997 SNPs throughout the genome, we document a weak genetic structure over the large scale, potentially indicative of high connectivity with extensive gene flow. No differentiation was detected at the smaller scale. We find indications of isolation‐by‐distance both for relative (*F*_ST_) and absolute divergence (Dxy). The two northernmost populations are most differentiated from the remaining populations, and diverge in parallel to the southern populations for a set of outlier loci. In conclusion, the population structure of *I. typographus* in Sweden is weak, suggesting a high capacity to disperse and establish outbreak populations in new territories.

## Graphical abstract

We investigated the genetic structure of the Eurasian spruce bark beetles over 800 km in Sweden using Restriction site associated DNA yielding thousands of markers across the genome. Our study show that population structure over this scale is weak, but we find indications of isolation by distance and that the Northern populations tended to be differentiated from the Southern ones.

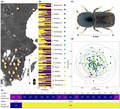

## 1 INTRODUCTION

The Eurasian spruce bark beetle, *Ips typographus* L., is one of the main disturbance agents in forests of mature Norway spruce (*Picea abies* L.) and other *Picea* spp. from Norway to Japan (Christiansen & Bakke, 1988). In periods of lower beetle population densities, this beetle is restricted to stressed or damaged trees, such as windthrows. However, during large outbreaks following extreme climatic events such as storms and severe drought (Marini et al., 2017), living, standing trees over vast areas may be affected (Komonen et al., 2011). Spruce bark beetle outbreaks have both ecological and economic consequences (Marini et al., 2013). In face of climate change, extreme weather events triggering bark beetle outbreaks are expected to increase (Jönsson et al., 2007), with potentially severe effects on spruce forests in Sweden and elsewhere. Due to its large ecological and economic effects, it is important to understand the ecology of this species to better inform forestry management efforts, such as predicting which regions may be at risk of dispersal from outbreak areas.

When *I. typographus* beetles attack living trees, they depend on simultaneous attacks by large numbers of conspecifics, enabling them to jointly overcome the defense of the trees. Once having found and penetrated a suitable host tree, males rapidly produce an aggregation pheromone, attracting conspecifics over distances (Schlyter et al., 1987). This aspect of its ecology means that during local outbreaks, very large numbers of beetles may be produced in spatially well‐defined spots (Kärvemo et al., 2014; Schroeder & Lindelöw, 2002), supplying the critical numbers of beetles to overcome the defense also of living and healthy spruces. This may result in very high levels of tree mortality (Kärvemo et al., 2014). The distances dispersed, and the frequency at which dispersal takes place, are crucial parameters for predicting the area under risk of spread of outbreaks. Most spruce bark beetle attacks are estimated to occur within 500 meters from the current outbreak (Wichmann & Ravn, 2001). However, dispersal distances of several kilometers have been estimated from mark‐recapture studies (Weslien & Lindelöw, 1990). Dispersal distances, especially rare dispersal events over long distances, are typically extremely challenging to monitor using standard ecological techniques such as mark‐recapture (Doležal et al., 2016; Duelli et al., 1997; Helland et al., 1989; Schlyter, 1992; Weslien & Lindelöw, 1990; Zolubas & Byers, 1995). As rare long‐distance dispersal can be a first step toward new outbreaks, novel methods for monitoring such dispersal and hence the potential for building up the numbers of individuals needed to cause outbreaks are thus needed (Li et al., 2021).

As genetic sequencing methods have improved, they have become an increasingly important tool to estimate gene flow and connectivity between populations (Dickson et al., 2019; Spear et al., 2010). While the exact distances and numbers of individuals cannot be monitored, genetic clustering methods provide insights into the spatial structuring of genetic variation, taking dispersal into account (Lawson et al., 2012). There are also genetic methods for inferring the effect of habitat type and distances on connectivity, estimating the dispersal resistance for all possible dispersal routes (Dickson et al., 2019). Such methods have the potential to inform management of which areas are at risk of receiving dispersing individuals from identified outbreaks.

Most previous genetic studies in *I. typographus* have used small sets of markers, for example, microsatellite or mitochondrial DNA. As each microsatellite loci needs to be individually identified, a number of markers of the same magnitude as those produced by, for example, restriction site‐associated DNA sequencing (RADseq) is very rarely available, limiting the power to detect finer population structure (Mayer et al., 2015; Putman & Carbone, 2014; Sunde et al., 2020). Moreover, their application to estimate standard population genetic parameters including *F*_ST_, detect clustering, and estimate effective population sizes all have important weaknesses (Putman & Carbone, 2014). The commonly used mitochondrial DNA markers reflect maternal inheritance only, are not recombining, and may be biased due to selection by maternally inherited symbionts (Hurst & Jiggins, 2005). Moreover, there may be discrepancies between nuclear and mitochondrial DNA (Shaw, 2002). A previous microsatellite‐based study detected little genetic structure with only two main clusters at the continental European scale (Mayer et al., 2015), while a study based on mitochondrial DNA found evidence consistent with colonization from both a southern and a northeastern refugia, but little diversity within Sweden (Mayer et al., 2014).

RADseq data have the potential to improve the resolution at which population structure can be detected, and in the present study, we apply this technique to pheromone trap samples of populations of *I. typographus* in Sweden, distributed along ca. 830 km, from Strömsund in the north to Nyteboda in the south, to evaluate the population structure of the Swedish population and investigate factors affecting connectivity. The ultimate goal is to improve our understanding of population structure and dispersal patterns of *I. typographus* to inform management of spruce forests in Sweden and elsewhere.

## 2 MATERIALS AND METHODS

### 2.1 Material and sampling

**FIGURE 1:**
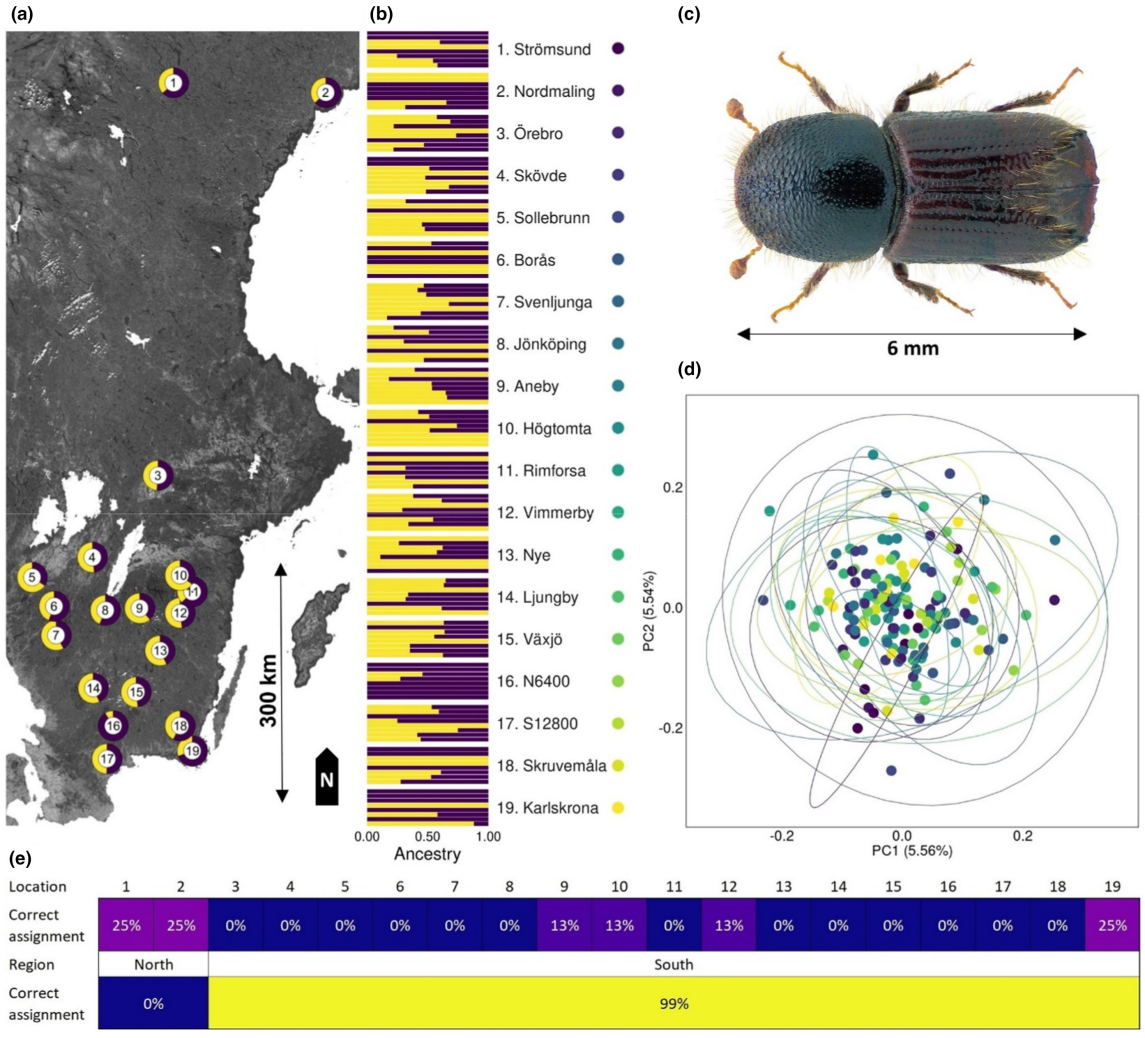
(a) Spatial distribution of sampling locations in Sweden with pool ball charts representing mean *K* = 2 assignment from ADMIXTURE and sampling location id in the center. (b) While one cluster (*K* = 1) was the most parsimonious explanation, we here illustrate the best grouping when individuals are assigned to two clusters (*K* = 2) using ADMIXTURE to shed light on the lack of population structure. The probability of belonging to each cluster is reported per sampling location with sampling location id and name (N6400 and S12800 correspond to localities north and south of Nyteboda). (c) Mature adult of *Ips typographus* (picture retrieved from https://commons.wikimedia.org/wiki/File:Ips_typographus_(Linn%C3%A9_1758)_(32,558,007,471).png). (d) Principal components 1 and 2 from the PCA with ellipses encircling all individuals from each sampling location. Sampling location color id is given to the left of the graph. (e) Number of individuals correctly assigned to their original sampling location and region from the Linear Discriminant Analysis with leave‐one‐out cross‐validations

The *I. typographus* specimens were captured at local stations that were part of the Swedish Forest Agency and Södra (Sweden's largest forest owner association) networks. The stations were distributed throughout Sweden, and samples were gathered during the peak activity period in the spring of 2019 (Figure 1a). The large‐scale sampling spanned 19 main locations, ranging from Strömsund in the north to Nyteboda in the south (Table S1). These two locations are ca. 830 km apart. As new attacks by spruce bark beetles have typically been suggested to be within 500 m of previous attacks (Wichmann & Ravn, 2001), we additionally designed a transect to resolve population structure at a more local scale. Both transects started at a recent outbreak locality, Nyteboda forest, and stretched toward the north and the south, respectively. The north–south direction was chosen to reflect the main direction in the larger data set. The trap intervals started at 200 m and were doubled for each trap, resulting in a series of distances ranging between 200 m up and 13 km between each pair of traps in the south and 26 km in the north, with a cumulative maximum distance of 76 km between the southernmost and northernmost traps (Figure S1), thus an order of magnitude shorter than the larger scale.

Bark beetles were captured using Theysohn pheromone slit traps (Galko et al., 2016) with a ca. 100 m expected sampling range (Schlyter, 1992), collected within a week during April 2019, and stored in 96% ethanol and frozen as they arrived to Lund University. Traps from the transect were emptied the day after the trap was set up and only living individuals were preserved at −80°C. Traps from the larger‐scale swarming surveillance were emptied within a week, but differences in the time the individuals had been dead prior to being stored in ethanol implied that DNA quality could vary across stations. We sampled 10 individuals per population and transect location, but included fewer specimens in a few populations where the DNA was degraded (Table S1).

### 2.2 DNA extraction and library preparation

The DNA extraction protocol, the RADtag library protocol, and summaries of the RADtag library preparation have been deposited in a public repository (https://github.com/sjellerstrand/ips_typographus_genomics). DNA was extracted using the Qiagen blood and tissue kit, following the protocol suggested for insect DNA. The whole insect was homogenized with the TissueLyser for 4 min at 30 Hz. The final DNA elution was done in 60 μl buffer EB. We monitored DNA concentration with a NanoDrop, and performed Qubit analyses on a subset of the samples (14 out of 634 extracted samples; four of these samples were among the 320 that were sequenced) as nanodrop quantification may inflate the estimated concentration. To further assess DNA quality, all extractions were run on agarose gels to estimate fragment size. Samples with concentrations below ~13 ng/μl or smeared profiles on the gel, indicative of decomposed DNA, were omitted from further analyses.

For the two transects, two single‐digest RADtag libraries were created from 160 samples per library. All samples from each transect were pooled in the same library to avoid any cross‐library batch effects. Of the 320 samples, 13 constituted individual duplicates originating from the same extract, and were split into two different tubes and treated as separate samples before library preparation to enable us to assess the repeatability of the method. From each sample, 500 ng DNA was used for restriction digest with the restriction enzyme SbfI from New England Biolabs. The DNA was sheared to allow for identification and removal of PCR duplicates. Individual barcoding was performed with 40 P1 inline barcodes of 8 bp differing by at least three sequence positions, combined with four P2 index barcodes of 8 bp differing by at least six sequence positions. Both libraries were amplified with a 16 cycle PCR. We performed size selection using SPRI beads throughout the library preparation, with a final size selection following the PCR step. The bulk of the final library consisted of fragments in the size range of 380–730 bp. The RADtag libraries were sequenced on separate lanes on an SP flow cell as paired‐end 150 bp reads on an Illumina NovaSeq 6000 platform by National Genomics Infrastructure – Stockholm, Sweden (NGI Stockholm, https://www.scilifelab.se/facilities/ngi‐stockholm/ http://ugc.igp.uu.se/our‐services/ngs‐technologies/).

### 2.3 RADseq data filtering and variant calling

All the codes for RADseq data filtering, variant calling, and population genomic analyses have been deposited in a public repository (https://github.com/sjellerstrand/ips_typographus_genomics). The RADseq data were demultiplexed by the NGI sequencing facility to sublibraries based on the index barcodes on the P2 adapter. These libraries were then demultiplexed with the bioinformatics pipeline Stacks 2.53 (Catchen et al., 2011; Catchen et al., 2013; Rochette et al., 2019), rescuing barcodes with a maximum of one mismatch, and filtering adapter sequences allowing for two mismatches. PCR duplicates were then removed based on the randomly sheared paired‐end reads, and adapters were removed using Trimmomatic 0.36 (Bolger et al., 2014), by providing custom adapter lists. Read quality was evaluated with FastQC 0.11.8 (https://www.bioinformatics.babraham.ac.uk/projects/fastqc). The reads were aligned to a concatenated reference genome consisting of the nuclear genome assembled by Powell et al. (2021) and the mitochondrion assembled by Lv et al. (2017) using BWA‐MEM 0.7.17 (Li & Durbin, 2009). After alignment, the bam files were sorted by coordinates with SAMtools 1.10 (Li et al., 2009) and only the single‐end reads were extracted for variant calling.

Variants were called with HaplotypeCaller from GATK 4.1.4.1 (McKenna et al., 2010) using a minimum base quality of 20 from non‐soft clipped bases. To correct for low levels of sequencing errors, batch effects, and cross‐contamination, we filtered out minor alleles representing <10% of the reads per individual and site. The resulting gVCF files were combined with CombineGVCFs and genotypes were called using GenotypeGVCFs on properly paired reads with a minimum phred‐scaled confidence of 20, and then SNPs were extracted. The assembled reference genome contains a large portion of repetitive sequences and low complexity regions, which were hard masked in the assembly (Powell et al., 2021). VCFtools 0.1.16 (Danecek et al., 2011) was used to filter out the hard masked regions. Briefly, we filtered SNPs according to GATK hard filtering practice, and for a minimum quality of 30, minimum read depth of 5, as well as additional filters following O'Leary et al. (2018), which are specified in Table S1. Samples with low coverage, high missingness, or evidence of severe cross‐contamination were removed from the dataset as follows: genotypes with a depth below 30, sites with a mean depth less than 48, a mean depth higher than 52, sites present in less than 80% of the individuals were filtered out.

The error rate of the dataset was evaluated for seven duplicates samples using BCFtools gtcheck with genotype likelihoods and used to examine the error rate, and we determined filtration criteria based on this rate. Duplicate samples were then removed from the dataset. To obtain balanced sample sizes, we sampled eight individuals per sampling location, with only seven individuals in two of the transect locations, as we did not have eight individuals for these. Variants present in less than five individuals in any sampling location were removed, as well as sites present in <90% of the remaining individuals.

We divided the samples into one dataset containing all the regional locations and two of the transect locations, and a second data set containing the transect locations only. We kept only alleles still present at least 3 times in each new dataset. Plink 1.90b4.9 (Purcell et al., 2007) was used to linkage prune the data using 50 kb windows, step sizes of 10 kb and a cut‐off at a r‐squared value higher than 0.1 (see Table S1 for number of SNPs following each filter step). Due to the absence of genetic structure in the shorter, local transect dataset, only the larger, regional dataset was used and reported for all subsequent analyses. To evaluate whether the conservative filtering approach, aimed to reduce the error rates between the duplicates included to check the quality of our calls, a less stringent filtering approach was applied to the regional dataset for comparison resulting in 3398 SNPs for the linkage pruned data set. We found no differences between the two data sets (Table S1, data not shown).

### 2.4 Population genomic analyses

As a first examination of populations structure, we performed a principal component analysis (PCA) as implemented in Plink 1.90b4.9 (Purcell et al., 2007) on the linkage pruned dataset. After visual inspection of eigenvalue decay using a broken‐stick assessment, the first four principal components were deemed significant (Figure S2). These were extracted and used as response variables in two multivariate analyses of variance (MANOVA) with the dependent variable set as either the sampling location or the region (the two northernmost sampling locations were merged into a northern region and all southern sampling locations into a southern region). The partial *η* ^2^ was calculated with the R‐package heplots 1.3‐5 (Fox et al., 2009) and used as a measure of effect size. To estimate the most parsimonious number of clusters in the data and assign populations to these, an admixture analysis was performed. The clustering was performed using ADMIXTURE 1.3.0 (Alexander et al., 2009) for *K* ranging from 1 to the 19 sampling locations and 100 cross‐validations for estimating the best *K*. As an additional test of clustering, we used fineRADstructure (Malinsky et al., 2018). A pairwise coancestry matrix of haplotype information was created from the nonpruned dataset with RADpainter 0.2 (Malinsky et al., 2018) and analyzed for population structure using fineSTRUCTURE 4.0.1 (Lawson et al., 2012) with 10,000,000 burn in iterations. During the 10,000,000 sample iterations output was recorded every 10,000 iteration, resulting in 1,000,000 tree comparisons. Finally, as an additional way to assess if there was evidence for population structure, we evaluated to which extent sampling locations could be discriminated based on the individual genotypes. This was done by performing a Linear Discriminant Analysis (LDA) on the same principal components and with either sampling locations or regions with the R‐package MASS 7.3–51.6 (Venables & Ripley, 2002), using leave‐one‐out cross‐validations for posterior assignment probabilities.

To assess the level of diversity for each locality, observed heterozygosity per site was calculated on the linkage‐pruned dataset. On the dataset not pruned for linkage, an estimate of the variation within each population, the mean nucleotide diversity was calculated in a sliding window of 100 kb in size with 25 kb steps. To identify regions under different selection pressures, Tajima's D, an allele frequency spectrum‐based estimate of selection, was calculated in a sliding window of 100 kb in size using VCFtools. Regions with a lower value of Tajima's D compared to the genome wide average are likely to have been under directional selection, whereas regions with a higher value are likely to have been under balancing selection (Nielsen, 2001). Private alleles were retrieved with POPULATIONS from Stacks as a complement to assess population structure. Pairwise divergence was estimated using the nonpruned regional dataset. Differentiation was estimated as global Weir and Cockerham's Weighted *F*_ST_ using VCFtools, absolute divergence Dxy was estimated using the python script popgenWindows.py (https://github.com/simonhmartin/genomics_general), and Euclidean distances were calculated from the first four principal components using Plink 1.90b4.9 (Purcell et al., 2007).

We assessed isolation by distance by performing Mantel tests based on the pairwise statistics estimates. Pairwise geographic distances were estimated using the R‐package Raster 3.1–5 (https://github.com/rspatial/raster). Weighted *F*_ST_ was transformed to *F*_ST_/(1 − *F*_ST_) following Rousset (1997). The geographic distances, absolute divergence Dxy, and Euclidean distances were all log_10_‐transformed. Each genetic measure was then analyzed against the pairwise geographic distances using one‐sided Mantel tests implemented in the R‐package ade4 1.7.15 (Dray & Dufour, 2007), using 1,000,000 permutations. The sampling location of Rimforsa was excluded from these analyses, as it is located very close to Högtomta and would mean doubling the sample size for what molecularly would be one location.

To identify regions of divergence and signals of selection between the southern sampling locations and the two in northern Sweden, pairwise Weighted *F*_ST_ and absolute divergence Dxy was calculated in genomic windows as described above, as well as Tajima's D, and Nucleotide Diversity including all sampling locations from the regional dataset. Outlier regions, above the upper 95 and 99 quantiles, were extracted. An additional *F*_ST_ based outlier analysis was employed using OutFLANK 0.2 (Whitlock & Lotterhos, 2015). OutFLANK was run with default parameter settings, extracting outlier with an expected heterozygosity higher than 0.1, and a q‐threshold lower than 0.01. All annotated genes within the top outlier windows were extracted from the recently published annotated genome of the species (Powell et al., 2021).

## 3 RESULTS

### 3.1 RADseq data summary

The two libraries produced 954.8 million raw read pairs. After demultiplexing, removal of PCR duplicates, adapters and quality trimming, and alignment to the reference genome (see Table S1 for details), on average 0.303 million quality single‐end reads remained per sample (± 1.80 SD, range 0.148–1.08). The aligned single‐end reads were evenly spread across the genome, and estimated to cover ca. 0.8% of the entire genome (±0.31 SD).

After variant calling and filtering, 2391 SNPs were retrieved for all 250 individuals (plus the seven duplicates). The error rate after filtering was estimated to 1.25% using the seven duplicate pairs (±0.36 SD, range 0.76–1.98). After removing duplicates and down sampling of the dataset, the regional dataset contained 1997 non‐pruned and 1409 pruned SNPs from 152 individuals and 19 sampling locations. The final average sample coverage for the regional non‐pruned dataset was 49× (±0.30 SD, range 49–50; See Table S1 for specifications).

### 3.2 Population genomic analyses

We found no evidence for population structure of *I. typographus* within the sampled range as both an exploratory PCA (Figure 1d), and the admixture analyses (Figure 1a,b) show that none of the populations are genetically divergent. The most parsimonious number of clusters estimated using cross‐validation error was 1 (one) (Figure S3). For illustration purposes, we present the assignment into two clusters as per sampling location averages throughout the geographic range (Figure 1a) and per individual as barplots (Figure 1b). A MANOVA based on the first four principal components of the PCA only recovered weak support for structure between sampling locations (*F*_18,133_, *p* = .10, *η* ^2^ = 0.14), with the third principal component driving the trend (*F*_18,133_, *p* = .025), and weak support between the northern and southern regions (*F*_1,150_, *p* = .075, *η* ^2^ = 0.056), with the second principal component driving the trend (*F*_1,150_, *p* = .0063). The linear discriminant analysis of sampling locations has an accuracy of 0.059 in assigning an individual to its original sampling location. The assignment to southern and northern regions was higher (accuracy of 0.89), but this was an artifact of the individuals assigning correctly to the southern region with much higher number of individuals (Figure 1e). Consistent with these findings, the fineRADstructure analysis was not able to cluster individuals from the same sampling locations (Figure S4).

**FIGURE 2:**
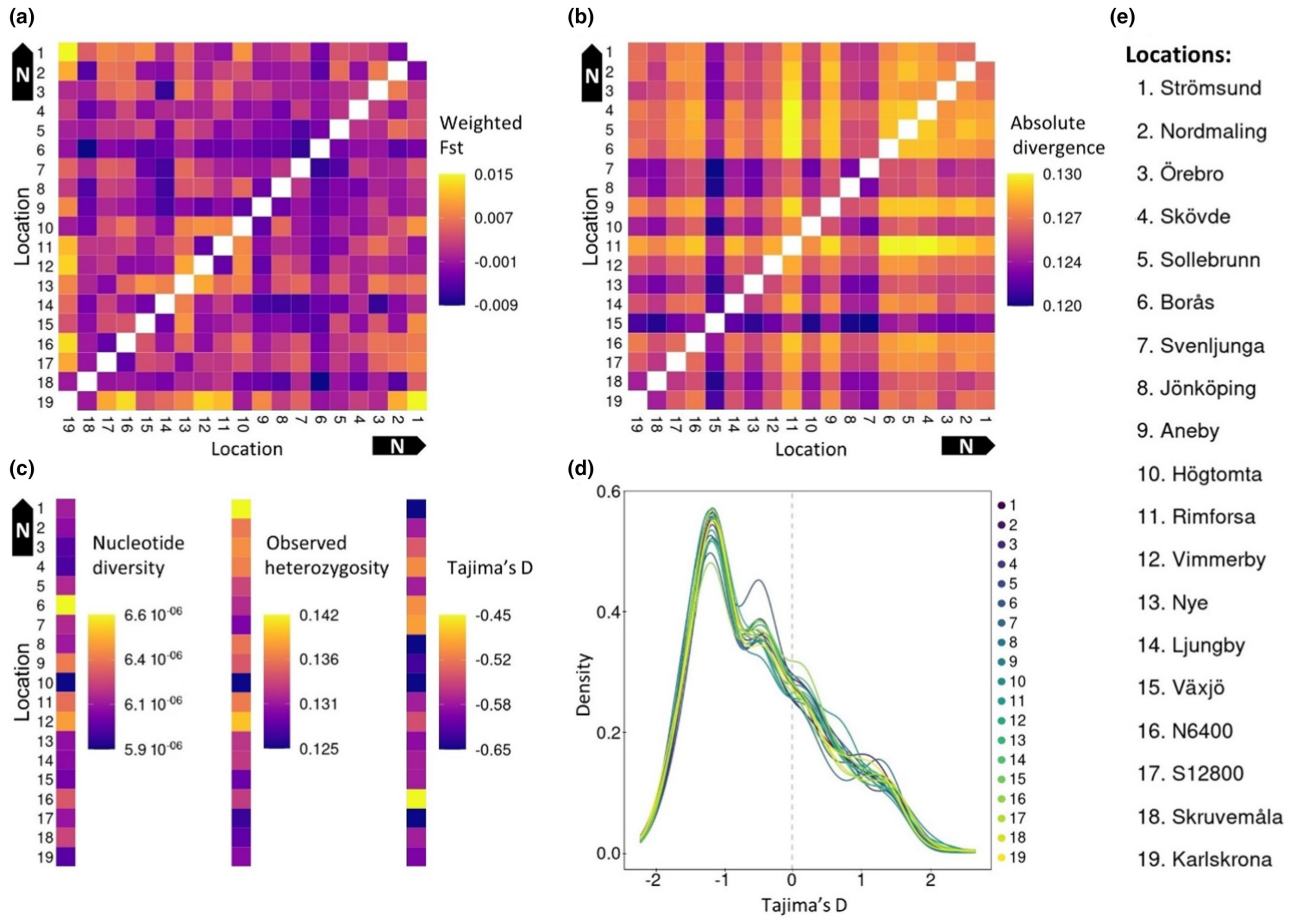
Pairwise and per sampling location population statistics reported in a northern to southern gradient. (a) Pairwise weir and Cockerham's weighted *F*_ST_. (b) Pairwise absolute divergence Dxy. (c) Per population nucleotide diversity, observed heterozygosity and Tajima's D. (d) Per sampling location probability density plot of Tajima's D, an allele frequency base measure of the demographic history of populations. Sampling location color id is given in the legend to the right of the graph. (e) Sampling location legend (N6400 and S12800 correspond to localities north and south of Nyteboda)

The pairwise differentiation, *F*_ST,_ confirmed the findings of a weak population structure, except for Karlskrona which, albeit not significant, appears slightly more differentiated from the other sampling locations (Figure 2a). The pairwise absolute divergence Dxy, showed that the southern sampling locations are more similar to each other, while the two sampling locations from Northern Sweden (Strömsund and Nordmaling), as well as some sampling locations in the northern part of Southern Sweden (Rimforsa, Aneby, Borås, Sollebrun, Skövde and Örebro) show evidence suggestive of weak, non‐significant divergence from other locations (Figure 2b). The pairwise Euclidian distances based on the first four principal components suggest low population structure, except for the population in the southeastern corner, Karlskrona that diverges somewhat from the other sampling locations (Figure S5). The per sampling location population statistics are similar across locations, consistent with low population structure. The two northern sampling locations have a higher level of observed heterozygosity, consistent with a higher effective population size, though (Figure 2c). We find negative estimates of Tajima's D across all sampling locations (Figure 2d), consistent with a recent population expansion. Finally, we found no private alleles in any of the sampling locations.

**FIGURE 3:**
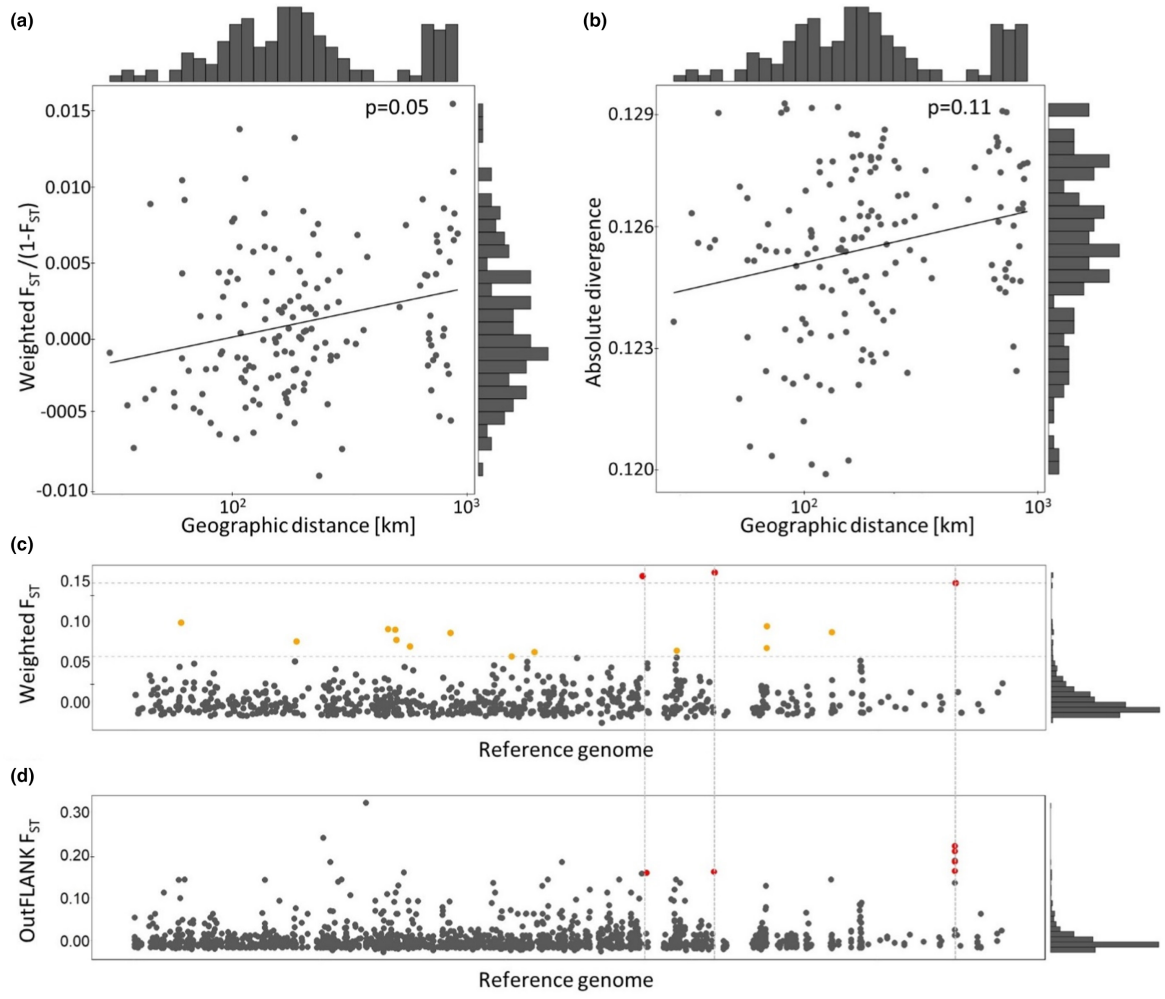
The effect of geographic distance on genetic differentiation illustrated as isolation by distance with (a) pairwise *F*_ST_ transformed to *F*_ST_/(1 − *F*_ST_), and logarithmized pairwise geographic distances, and (b) logarithmized pairwise absolute divergence Dxy and geographic distances. (c) Genomic regions that are strongly differentiated between the two northernmost and southern populations based on sliding window *F*_ST_, with 99% outliers depicted in red and 95% outliers in yellow. (d) Genomic regions that are strongly differentiated between the two northernmost and southern populations based on OutFLANK *F*_ST_, with expected heterozygosity higher than 0.1 and a q‐threshold lower than 0.01 depicted in red. As the genome is contig based, the contigs have not been colored, but are ordered from left to right by contig number

The Mantel tests of pairwise genetic distances against geographic distances suggest weak isolation by distance. While there was a marginally significant positive effect of distance on *F*_ST_ (*r* = 0.25, *p* = .05), absolute divergence Dxy did not increase significantly with distance (*r* = 0.24, *p* = .11), and pairwise genetic differentiation of Euclidean distances was borderline significant (*r* = 0.21, *p* = .05, Figure 3a,b; Figure S6).

We then examined the genomic distribution of differentiation to address if specific genomic regions (windows) were differentiated between the northern populations that were most strongly differentiated from all other populations and the entire group. For *F*_ST,_ we found 15 outlier windows between these regions, whereof three windows above the 99% quantile and 12 above the 95% quantile; Figure 3c; Table S1). The midpoint coordinates of the three outlier windows above the 99% quantile were all located within annotated genes potentially under divergent selection, tyrosine‐protein kinase‐like otk (Ityp12817), PREDICTED: roquin‐1 (Ityp15302), and Down syndrome cell adhesion molecule‐like protein Dscam2 (Ityp21085). The outlier analysis of absolute divergence Dxy between the southern and the two northern sampling locations returned 13 coherent outlier regions from 49 windows above the 95% quantile, including 3 regions from 12 windows were above the 99% quantile (Figure S7A; Table S1). The results remain extremely similar when using OutFLANK for outlier detection (Figure 3; Table S1). These regions can potentially harbor variants that are important for adaptation and would be interesting to examine in a study including more northern populations.

We also examined if there were specific genomic regions showing indications of being under selection, and if outlier regions under stronger selection also were more differentiated. Across the entire dataset, we found elevated nucleotide diversity for 15 coherent outlier regions from 52 windows above the 95% quantile, whereof three regions from 11 windows were above the 99% quantile (Figure S7B, Table S1). The midpoint coordinate of the strongest outlier was located within an annotated gene and surrounded by outer 99% quantile outliers. It is described as serine/threonine‐protein kinase MARK2‐like isoform X17 (Ityp12430). Outliers indicative of different selection pressures, as estimated by Tajima's D for the entire data set, returned 13 coherent outlier regions from 13 windows above the 95% quantile, whereof three regions from 3 windows were above the 99% quantile (Figure S7C; Table S1). The midpoint coordinate of the strongest outlier was located between two annotated genes. These are described as calpain‐A‐like isoform X1 (Ityp15176, 10 kb upstream) and PREDICTED: uncharacterized protein LOC109546387 (Ityp15175, 17.8 kb downstream).

While none of the strongest outliers overlap, the strong outlier of Tajima's D which reflects loci under strong selection on contig 18 overlaps with outliers of absolute divergence Dxy and nucleotide diversity above the 95% quantile, and is located 600 kb from one of the *F*_ST_ outliers above the 99%. The genes found in this region, include e.g. calpain‐A‐like isoform X1, involved in the organization of the actin‐related cytoskeleton during embryogenesis in *Drosophila*, PREDICTED: roquin‐1 gene and PREDICTED uncharacterized protein LOC109546387 (Table S1).

## 4 DISCUSSION

We here found strong evidence for pervasive gene flow among Eurasian spruce bark beetles throughout Sweden. While the reported dispersal distances during outbreaks typically are very modest (Weslien & Lindelöw, 1990), we found weak population structure over large geographical distances, which is consistent with both frequent dispersal and long‐distance dispersal events. This is in line with the findings of studies over continental Europe using lower resolution molecular markers (Mayer et al., 2015). Our high‐resolution molecular markers could not resolve a clear population structure, why we interpret the lack of genetic structure as resulting from pervasive gene flow. Potentially, the recent population expansion when Sweden was colonized by the spruce tree and beetle could also have contributed to the low divergence between populations. This study adds to the evidence for a large and well‐connected population of the Eurasian spruce bark beetle. The North American mountain pine beetle (*Dendroctonus ponderosae*) also has high connectivity, but a mountain range structures the population into two subpopulations (Janes et al., 2014).

Recently, Müller et al. (2022) performed a smaller study using genotype by sequencing for pooled samples from two sites 58 km apart in Central Europe, relying partly on our published genome (Powell et al., 2021). They conclude a high genetic variation in their two populations, but very low differentiation between the two. Thus, their findings support the conclusion based on our data set of 19 populations from Sweden sampled over a 10 times larger scale.

Moreover, at the much larger spatial scale of continental Europe, only two main clusters of *I. typographus* have been identified (Mayer et al., 2015). Therefore, large regions of unsuitable habitat may be needed for population structure to arise in some bark beetle species, consistent with findings of low differentiation in the large pine weevil *Hylobius abietis* (Conord et al., 2006). However, the poor disperser *Dendroctonus micans* shows a much stronger population structure within Europe (Mayer et al., 2015). Moreover, although it has the same host as *I. typographus*, the six‐toothed spruce bark beetle *Pityogenes chalcographus* harbors higher levels of genetic diversity, with post ice age colonization of older lineages from at least three different refugia (Bertheau et al., 2013). Importantly, we would have expected a clearer differentiation of our southern and northern populations given the evidence for the existence of mitochondrial differentiation among Swedish populations at this scale (Mayer et al., 2014). At least our two northernmost populations should belong to the northern cluster identified by Mayer and colleagues based on geographic proximity. While these two populations are the most strongly differentiated, they do not represent a discrete cluster. Potentially this discrepancy could be due to recombination being reflected in the nuclear genome but not in the mitochondrial genome (Mayer et al., 2014), as well as the lower effective population size of mitochondrial (25%) compared to nuclear loci. There could also be selection for specific mitochondrial haplotypes in different regions of Sweden. There is evidence for temperature dependent asymmetric introgression of mitochondrial DNA in the Eastern yellow robin (*Eopsaltria australis*; Morales et al., 2017) and a role for mitochondria in thermal tolerance in *Tigiropus* copepods (Harada et al., 2019), although these are very distantly related species. Experimental evidence is needed to disentangle the roles of colonization history and temperature tolerance in *I. typographus*. Finally, a potential rapid northward shift of the southern lineage may explain our findings. Unfortunately, the RADseq data only contain few mitochondrial markers with a lower coverage than expected compared to the nuclear markers, and we have therefore not analyzed these markers. Further analysis of mitochondrial DNA from our study populations is needed to understand this discrepancy.

Potentially, the present wide cover of spruce forest in most parts of Sweden might explain the general lack of population structure for nuclear data. Another, mutually non‐exclusive explanation, could be large‐scale triggering events such as storm damage or drought stress with large outbreaks and a high number of dispersing individuals that homogenize the genetic structure. A large scale northward expansion of populations with the southern genotype could explain the weak population structure recovered between northern and southern populations in our data, which contrasts the findings of two mitochondrial lineages in Sweden, one arriving from the northeast and one from the southwest (Mayer et al., 2014). Although the range covered by our study is similar to that in the Canadian and continental European studies, both recovering population structure, the continuity of the Swedish lowland spruce forest could have caused the weak structuring. One of the few populations that showed a tendency to differ despite not being separated by large distances, Karlskrona, is situated in the southeastern extreme of the Swedish distribution, a region where deciduous forests are more frequent. The lower proportion and connectivity of spruce forest in this region could have contributed to this trend. Data from larger areas of non‐spruce habitat are clearly needed to evaluate whether they may serve as barriers against dispersal in other geographic regions. Examining which habitat types that explain the reduction in gene flow enabling dominance of the northern mitochondrial haplotype in Norway, also at lower latitudes than in Sweden (Mayer et al., 2014), would be an interesting future investigation.

As there was a tendency for the two northernmost populations to be more differentiated, we further examined which genomic regions were differentiated between the northern and southern populations, and identified a few outlier regions. Potentially, selection for early maturation to reach the adult stage before winter could be stronger in the northern populations with shorter summers than the southern populations, as adult bark beetles may survive to −30°C (Annila, 1969), whereas eggs, larvae, and pupae have high mortality rates during winter (Annila, 1969; Baier et al., 2007; Faccoli, 2002). Further investigating the genes differing between northern and southern populations, and how they connect colonization routes is an interesting future line of research, as colonization from two different refugia, one northern and one southern is likely to have resulted in the population structure in *I. typographus* in Sweden (Mayer et al., 2014). These colonization routes are largely consistent with the colonization routes of the host plant, Norway spruce (*P. abies*; Mayer et al., 2014). In spite of the weak population structure found here, there is also a difference in diapause induction, with the northern populations typically being univoltine, while southern populations occasionally produce a second generation (Jönsson et al., 2009). A strong genetic component to voltinism was found in the two northernmost populations investigated by Schroeder and Dalin (2017). In populations where different strategies could be favorable different years, there could potentially be either selection for different strategies depending on the weather, gene flow re‐introducing alleles associated with bivoltinism from more southern populations, or genetic variants enabling a second generation to be plastically induced under the right conditions. Further research into the genetic underpinnings of voltinism is an interesting future direction.

An initial intention was to use the genetic data to address if specific habitats reduced dispersal of *I. typographus*. However, the lack of population structure means it is not possible to address how the densities of specific habitats affect dispersal probability, or to identify markers that could be used to assess the geographic origin of *I. typographus* individuals. Future studies of spruce bark beetle population structure should focus on even larger scales, geographically addressing the effects of larger water bodies, mountain ranges or larger areas of deciduous forests and agriculture on dispersal, but as well temporally the initial post‐glacial invasion of insect and host tree. Another intention was to identify region‐specific genetic markers, enabling the identification of the origin of individuals at new outbreaks (Shegelski et al., 2021). This is also not possible, given the very weak population structure. It might, however, be relevant to monitor more large‐scale dispersal of novel lineages or species, for example over water bodies, through timber transports or across mountain ranges, in the future to assess the potential for e.g. other species of bark beetles to invade new areas (Bentz et al., 2019). Moreover, as sequencing technologies are becoming cheaper, whole genome sequencing has the potential to further increase the resolution enabling even more fine‐scale studies of population differences, especially for species with small genomes such as *I. typographus*. Potentially, whole genome data could identify diverged regions that are not covered by our current RAD dataset. Including more populations from northern Sweden and performing whole genome sequencing that captures both mitochondrial and nuclear variants could also provide a more in depth understanding of the ecology of *I. typographus*.

In conclusion, our main finding is that population structure in *I. typographus* across a large regional scale in Sweden generally is weak or absent. A recent smaller study from Germany supports our results (Müller et al., 2022). A weak genetic structure is logically related to a high degree dispersal on several scales, which agrees with biological literature. Possibly, larger landscape scale outbreaks in one area could over time increase beetle population density of adjacent regions through an increased immigration to these areas. Any local effects of Global Warming (storms, severe drought stress, or fires) in such adjacent areas could then be potentiated by beetle population increases from external influx (Valeria et al., 2016). If, and how quickly, an increased population size in an outbreak area may spread through forest landscapes is an interesting topic for future studies.

## AUTHOR CONTRIBUTIONS

**Simon Jacobsen Ellerstrand:** Data curation (lead); formal analysis (lead); methodology (supporting); visualization (lead); writing – original draft (equal). **Shruti Choudhary:** Methodology (equal); writing – review and editing (equal). **Kajsa Svensson:** Data curation (equal); investigation (equal); methodology (equal). **Martin N. Andersson:** Resources (equal); writing – review and editing (equal). **Carsten Kirkeby:** Funding acquisition (equal); project administration (equal); writing – review and editing (equal). **Daniel Powell:** Formal analysis (equal); resources (equal). **Fredrik Schlyter:** Resources (equal); writing – review and editing (equal). **Anna Maria Jönsson:** Funding acquisition (equal); supervision (supporting); writing – review and editing (equal). **Mikkel Brydegaard:** Funding acquisition (equal); investigation (equal); methodology (equal); project administration (equal); writing – review and editing (equal). **Bengt Hansson:** Funding acquisition (equal); investigation (equal); methodology (equal); project administration (equal); supervision (supporting); writing – review and editing (equal). **Anna Runemark:** Conceptualization (lead); data curation (lead); formal analysis (equal); funding acquisition (equal); investigation (lead); methodology (lead); project administration (equal); resources (equal); supervision (lead); visualization (supporting); writing – original draft (supporting).

## CONFLICT OF INTEREST

We declare no conflicts of interest.

## Supporting information


Figure S1

Table S1

## Data Availability

The bam‐files containing all genetic information will be deposited on the European Nucleotide Archive https://www.ebi.ac.uk/ena upon publication. All scripts used for analyses are deposited on github https://github.com/sjellerstrand/ips_typographus_genomics and will be deposited on dryad upon publication.
