# Supplementary figures and images for: Weak population genetic structure in Eurasian spruce bark beetle over large regional scales in Sweden
Source: Ecol Evol. 2022 Jul 6;12(7):e9078. doi: 10.1002/ece3.9078 (PMC9260063; doi:10.1002/ece3.9078)

Figure S1
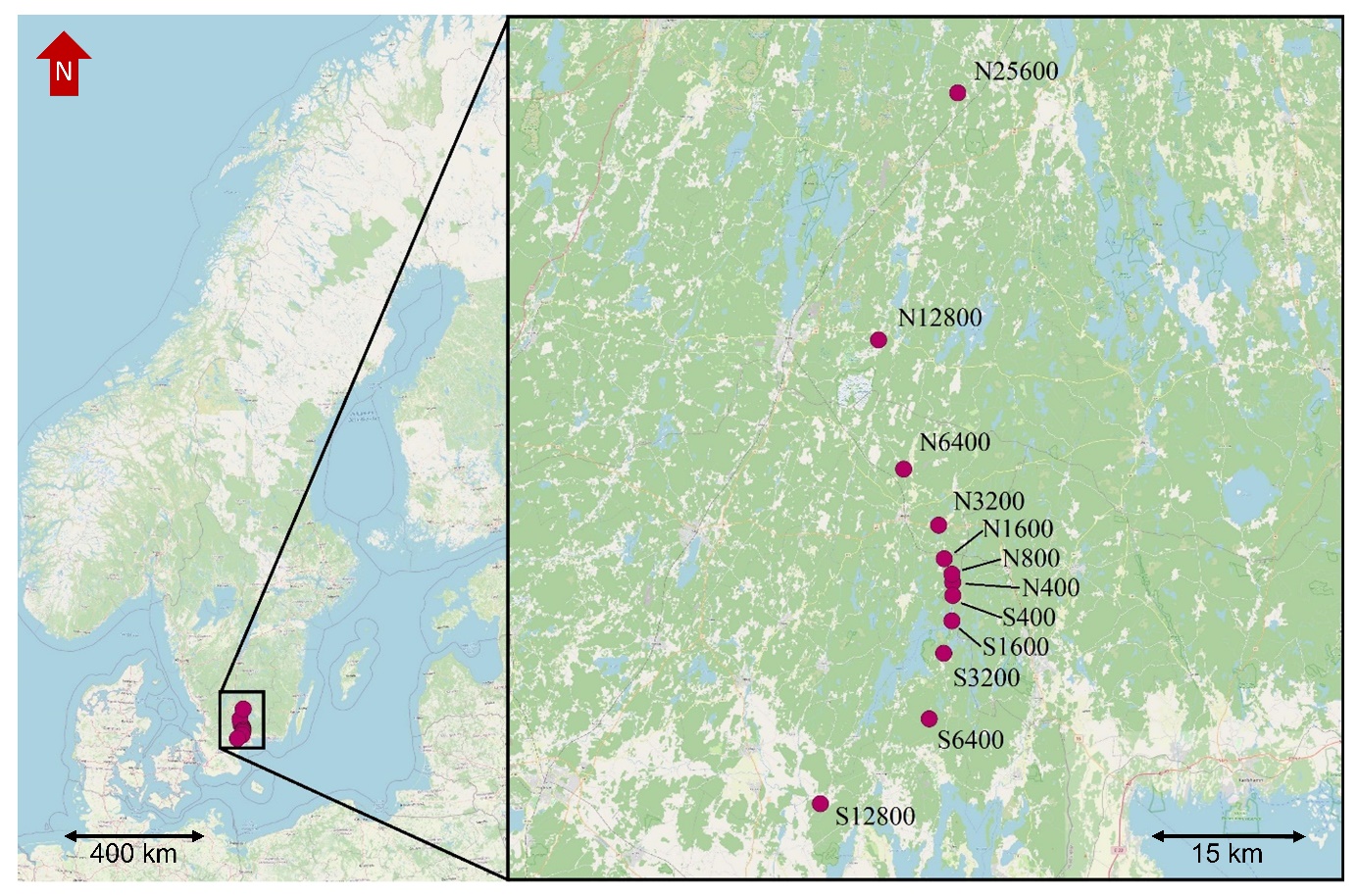


Figure S2

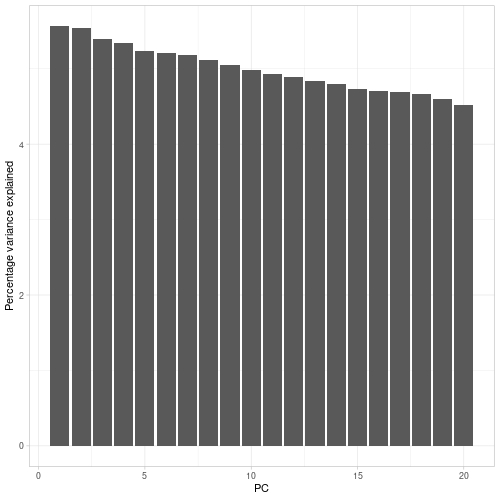


Figure S3

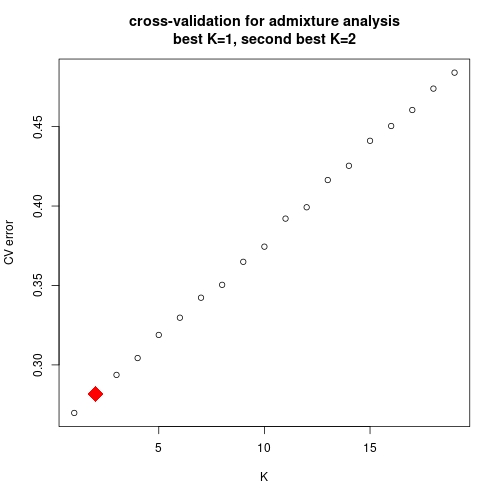


Figure S4
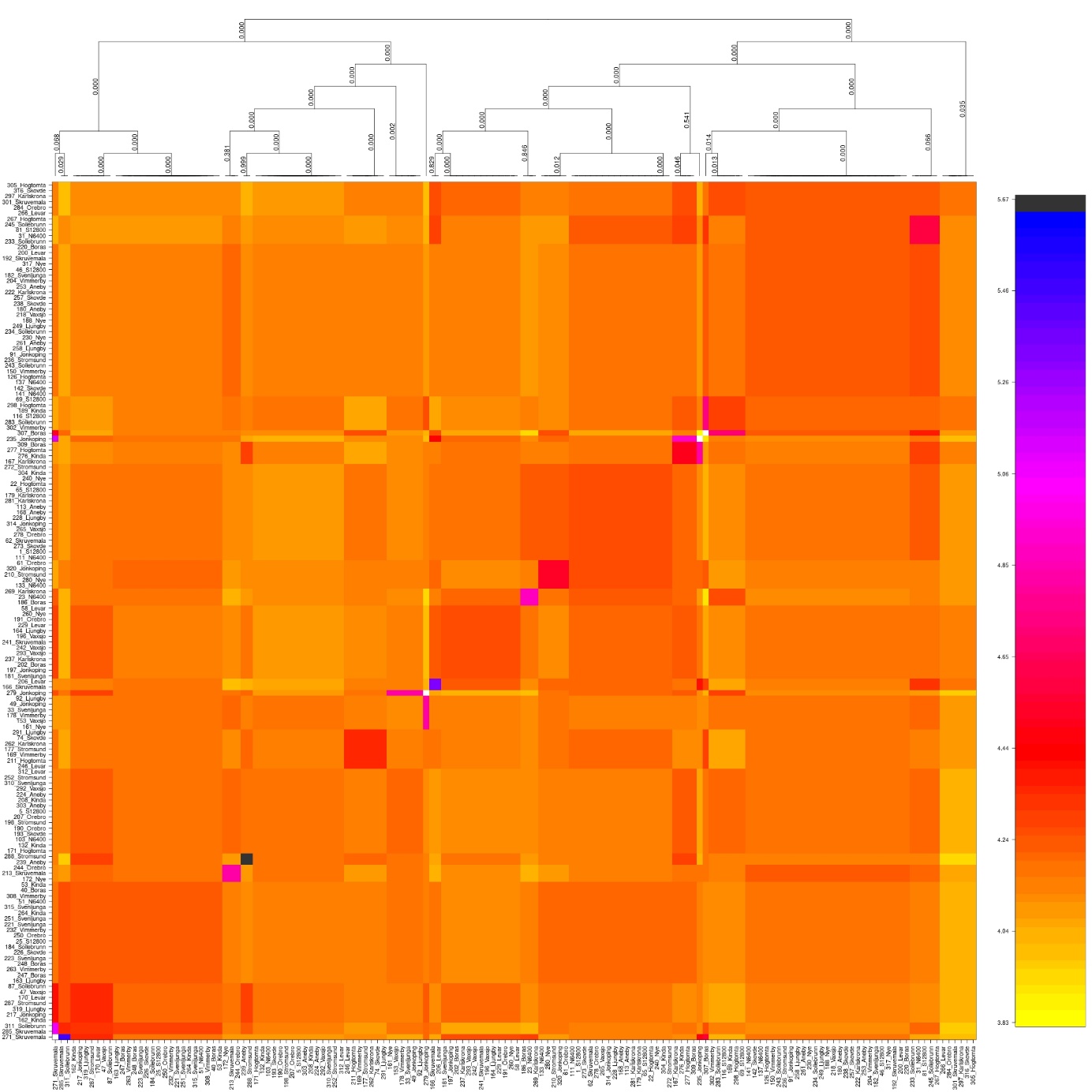


Figure S5

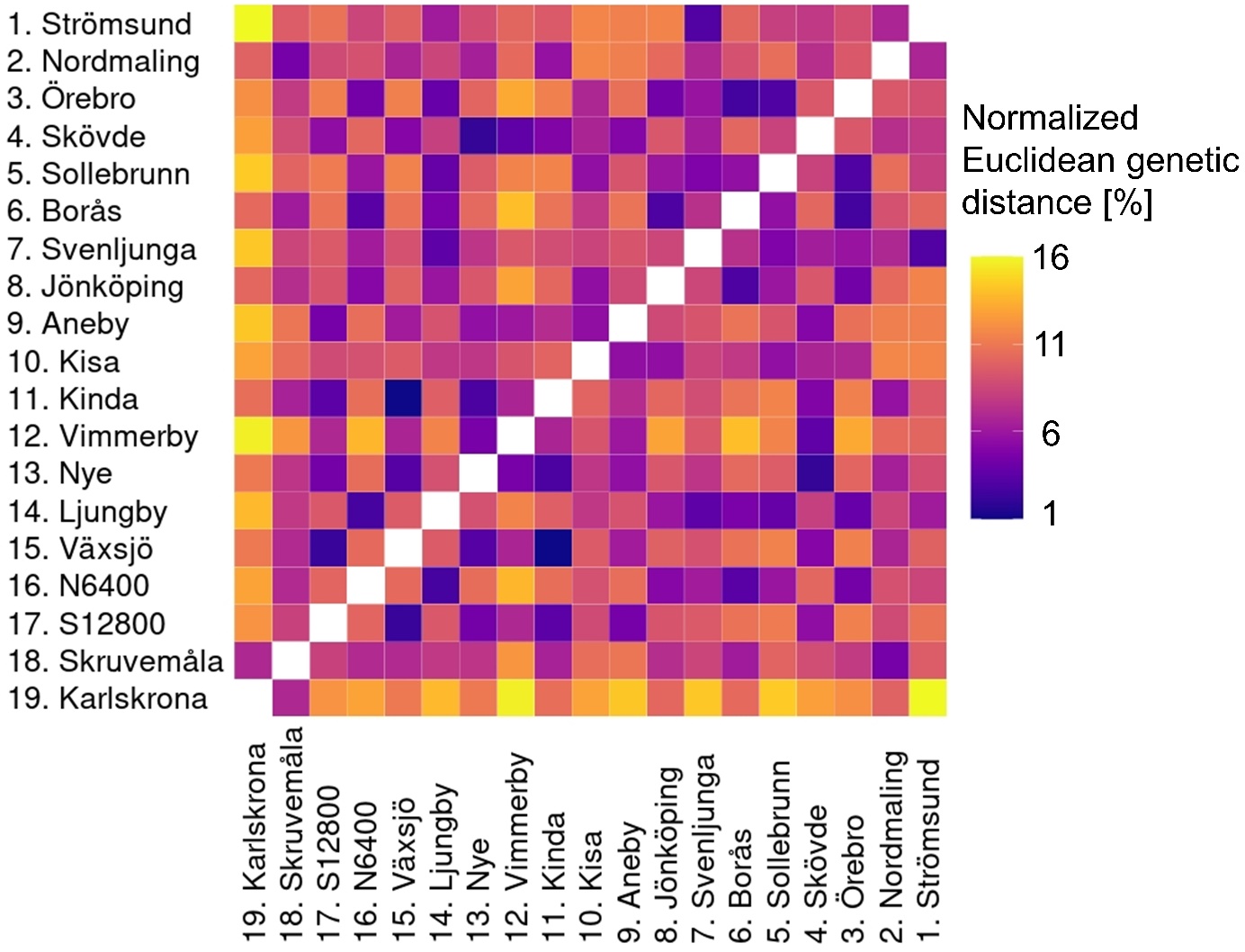


Figure S6


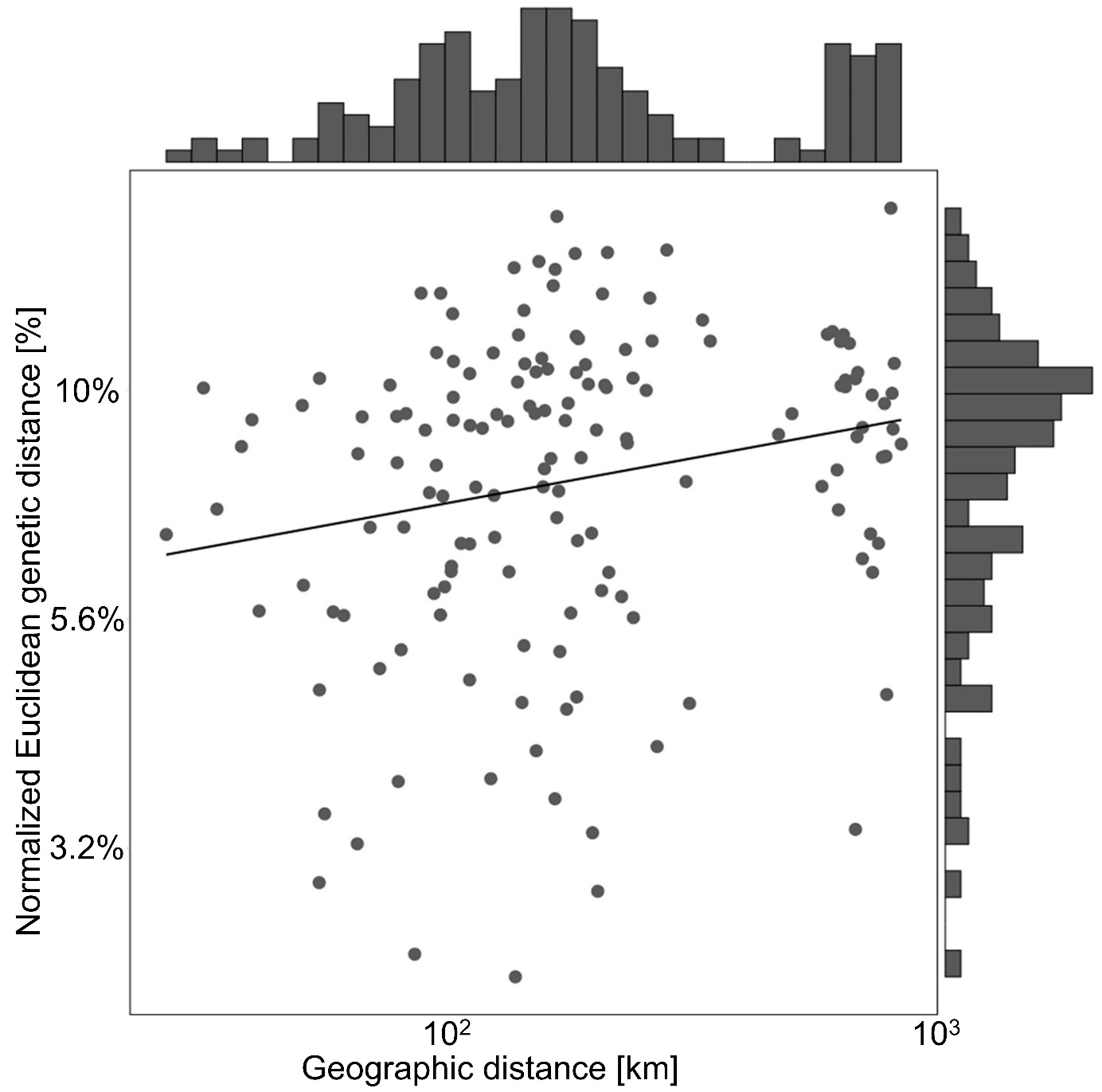


Figure S7


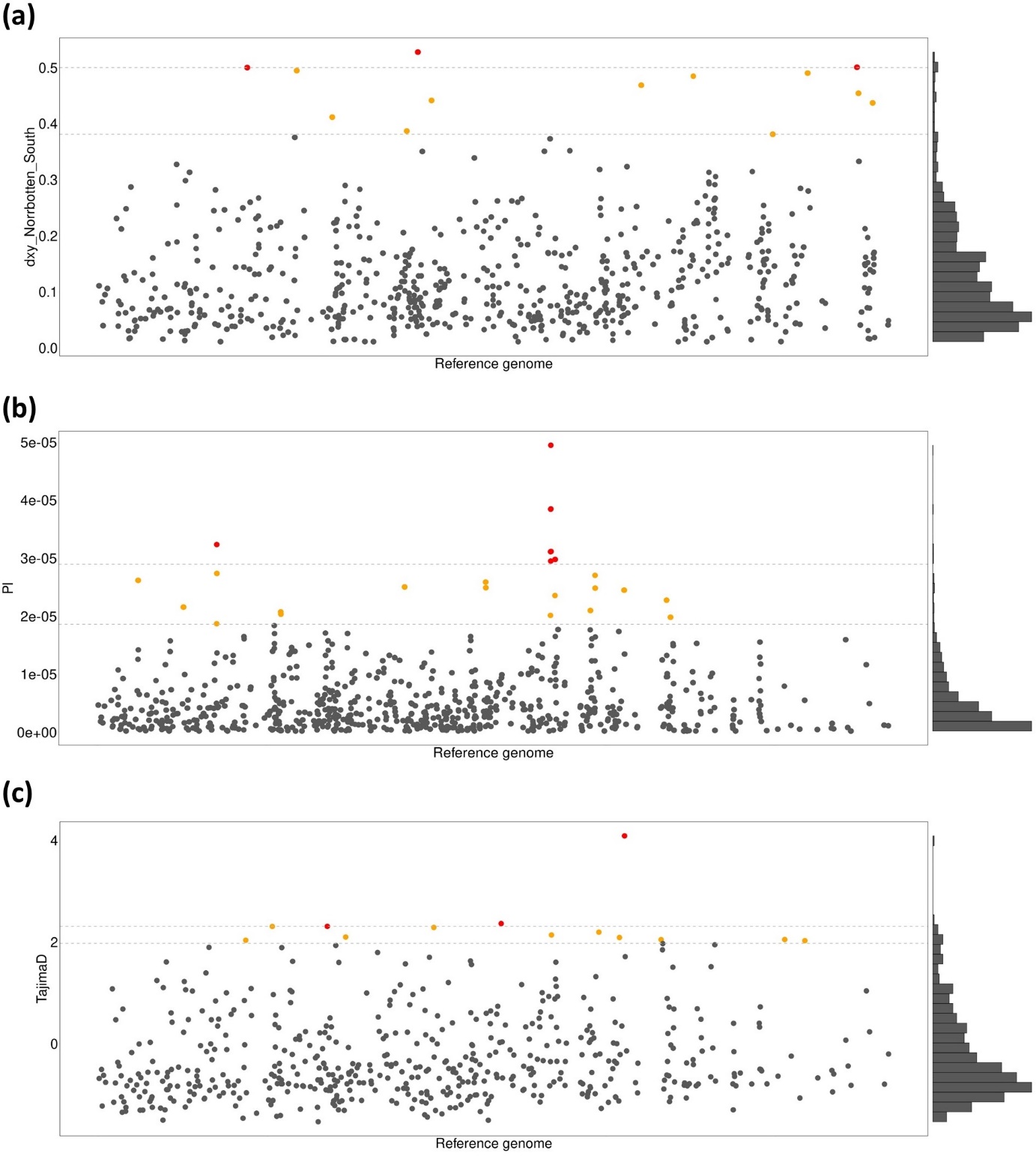

Supplement: Supplementary file 1 — Figure S1 [file ECE3-12-e9078-s002.docx]
