# Supplementary material for: Weak population genetic structure in Eurasian spruce bark beetle over large regional scales in Sweden
Source: Ecol Evol. 2022 Jul 6;12(7):e9078. doi: 10.1002/ece3.9078 (PMC9260063; doi:10.1002/ece3.9078)
Supplement: Supplementary file 2 — Table S1 [file ECE3-12-e9078-s001.docx]

# Appendix

**Table S1**. Sampling locations, their coordinates in SWEREF 99 with those extracted from the map using google maps marked “from maps”, and the number of samples.

| **Sampling location** | **Longitude** | **Latitude** | **Accurate coordinates** | **# Unique individuals** | **# Unique individuals in final dataset** |
| --- | --- | --- | --- | --- | --- |
| Aneby | 57.838367 | 14.822959 | From maps | 10 | 8 |
| Borås | 57.825047 | 13.009495 | From maps | 10 | 8 |
| Jönköping | 57.783019 | 14.16078 | From maps | 10 | 8 |
| Karlskrona | 56.23226 | 15.917688 | From maps | 8 | 8 |
| Ljungby | 56.908537 | 13.934731 | Yes | 10 | 8 |
| Högtomta | 58.176811 | 15.706293 | From maps | 10 | 8 |
| N12800 | 56.55068611 | 14.27779444 | Yes | 8 | 7 |
| N1600 | 56.361175 | 14.38385556 | Yes | 10 | 8 |
| N25600 | 56.76599167 | 14.39861667 | Yes | 9 | 8 |
| N3200 | 56.39028333 | 14.37478889 | Yes | 10 | 8 |
| N400 | 56.34077222 | 14.39740556 | Yes | 10 | 8 |
| N6400 | 56.43869444 | 14.31921944 | Yes | 10 | 8 |
| N800 | 56.34785 | 14.39615833 | Yes | 10 | 8 |
| Nordmaling | 63.588445 | 19.582435 | Yes | 10 | 8 |
| Nye | 57.343711 | 15.27246 | From maps | 10 | 8 |
| Olofström | 56.333165 | 14.649743 | From maps | 6 | 0 |
| Oskarström | 56.448838 | 13.323202 | From maps | 10 | 0 |
| Rimforsa | 58.1372 | 15.6830 | Yes | 10 | 8 |
| S12800 | 56.14729722 | 14.19501667 | Yes | 10 | 8 |
| S1600 | 56.30726389 | 14.3967 | Yes | 10 | 7 |
| S200 | 56.333275 | 14.39822222 | Yes | 7 | 0 |
| S3200 | 56.27905556 | 14.38444444 | Yes | 10 | 8 |
| S400 | 56.32934722 | 14.39775 | Yes | 10 | 8 |
| S6400 | 56.22196944 | 14.36273611 | Yes | 10 | 8 |
| Skruvemåla | 56.453803 | 15.703659 | From maps | 10 | 8 |
| Skövde | 58.390892 | 13.845809 | From maps | 10 | 8 |
| Sollebrunn | 58.120066 | 12.533529 | From maps | 10 | 8 |
| Strömsund | 63.768892 | 15.705972 | Yes | 10 | 8 |
| Svenljunga | 57.512252 | 13.12457 | Yes | 10 | 8 |
| Vimmerby | 57.801091 | 15.716988 | From maps | 10 | 8 |
| Växjö | 56.880893 | 14.803059 | From maps | 10 | 8 |
| Örebro | 59.32695 | 15.251517 | Yes | 9 | 8 |

**Figure S1**. Map of the transect locations. Note that due to discontinuity of the spruce forest habitat we did not place any S25600 trap.

**Figure S2**. Eigenvalue decay of principal components.

**Table S2**. Summary of applied VCF-filters, with resulting summary statistics following each filter step.

| **Pre-processing step of raw data** | **Program** | **Average number read pairs per individual** | **Remaining read pairs from previous step** | **Average SE read length** |
| --- | --- | --- | --- | --- |
| 0. Raw data |  | 2983604 |  | 151 |
| 1. Demultiplex data | Stacks 2.53 | 2517122 | 84.4% | 143 |
| 2. Remove PCR-duplicates | Stacks 2.53 | 1041599 | 41.4% | 143 |
| 3. Adapter and quality trim reads | Trimmomatic 0.36 | 842394 | 80.9% | 142.8 |
| 4. Align reads to reference | BWA-MEM 0.7.17 | 302581 | 72.5% |  |

**Table S3**. Summary of applied VCF-filters, with resulting summary statistics following each filter step.

| **Filter name** | **Program** | **Filter definition** | **Number of sampling sites** | **Number of individuals** | **Number of duplicates** | **Number of SNP sites** | **Average individual depth** | **Average error rate** |
| --- | --- | --- | --- | --- | --- | --- | --- | --- |
| 1. Extract SNPs | GATK 4.1.4.1 | Keep site if SNP | 32 | 307 | 13 | 473581 | 3.72 | 1.69% |
| 2. Minimum filters | VCFtools 0.1.16 | Keep site if max-missing=0.5, min-meanDP=10, minDP=5, minQ=30, and mac=3 | 32 | 307 | 13 | 17202 | 46.45 | 10.88% |
| 3. Remove masked sites | VCFtools 0.1.16 | Remove site if within repeat sequence or low complexity regions | 32 | 307 | 13 | 10736 | 48.49 | 10.67% |
| 4. GATK Hard filters | GATK 4.1.4.1 | Remove site if QD < 5.0, MQ < 45.0, MQRankSum < -5.0, ReadPosRankSum < -3.0, and ExcessHet > 54.69 | 32 | 307 | 13 | 7327 | 48.20 | 9.26% |
| 5. Total allelic balance per heterozygous site | BCFtools 1.10 + AWK +  VCFtools 0.1.16 | Keep site if AB > 0.28 and AB < 0.72, or if AB < 0.01, or if AB > 0.99 | 32 | 307 | 13 | 6529 | 48.88 | 8.93% |
| 6. Strand bias | BCFtools 1.10 + AWK +  VCFtools 0.1.16 | Keep site if SAF/SAR > 100 and SRF/SRR > 100, or if SAR/SAF > 100 and SRR/SRF > 100 | 32 | 307 | 13 | 6453 | 49.03 | 8.85% |
| 7. Remove some individuals and filter by depth | VCFtools 0.1.16 | Remove troublesome individuals, and keep site if max-missing=0.8, min-meanDP=48, minDP=30, max-meanDP=52, and mac=3 | 32 | 286 | 10 | 2458 | 49.49 | 2.53% |
| 8. Individual allelic balance per heterozygous site | allelicBalance.py* + VCFtools 0.1.16 | Recode site to homozygote if minor allele AB < 0.14, remove site if minor allele AB > 0.14 and AB < 0.28, and if mac=3 | 32 | 286 | 10 | 2391 | 49.49 | 1.25% |
| 9. Down sample dataset | VCFtools 0.1.16 | Remove duplicate individuals, down sample dataset, and remove site if max-missing=0.8, and mac=3 | 29 | 230 | 0 | 2317 | 49.50 |  |
| 10. Missingness per population | AWK +  VCFtools 0.1.16 | Keep site if present in at least 5 individuals in all populations, and if max-missing=0.9 | 29 | 230 | 0 | 2242 | 49.49 |  |
| 11. Create Regional dataset | VCFtools 0.1.16 | Keep individuals for the regional dataset, and remove site if mac=3 | 29 | 230 | 0 | 1997 | 49.74 |  |
| 12. Linkage prune | Plink 1.90b4.9 + AWK +  VCFtools 0.1.16 | Keep sites that are not in linkage | 19 | 152 | 0 | 1409 | 49.54 |  |

*modified from https://github.com/joanam/scripts/blob/master/allelicBalance.py

**Table S4**. Summary of applied VCF-filters for the less stringent dataset, with resulting summary statistics following each filter step.

| **Filter name** | **Program** | **Filter definition** | **Number of sampling sites** | **Number of individuals** | **Number of SNP sites** | **Average individual depth** |
| --- | --- | --- | --- | --- | --- | --- |
| 1. Extract SNPs | GATK 4.1.4.1 | Keep site if SNP | 32 | 307 | 473581 | 3.72 |
| 2. Hard filters and create Regional dataset | VCFtools 0.1.16 | Keep site if max-missing=0.8, min-meanDP=10, minDP=5, max-meanDP=75, minQ=30, and mac=3. Only keep individuals for the regional dataset | 19 | 152 | 11235 | 46.67 |
| 3. Remove masked sites | VCFtools 0.1.16 | Remove site if within repeat sequence or low complexity regions | 19 | 152 | 7261 | 47.64 |
| 4. GATK Hard filters | GATK 4.1.4.1 | Remove site if QD < 2.0, MQ < 40.0, MQRankSum < -12.5, ReadPosRankSum < -8.0 | 19 | 152 | 6429 | 48.46 |
| 5. Individual allelic balance per heterozygous site | allelicBalance.py + VCFtools 0.1.16 | Recode site to homozygote if minor allele AB < 0.14, remove site if minor allele AB > 0.14 and AB < 0.28, and if mac=3 and max-missing=0.8. | 19 | 152 | 5971 | 48.91 |
| 7. Linkage prune | Plink 1.90b4.9 + AWK + VCFtools 0.1.16 | Keep sites that are not in linkage. | 19 | 152 | 3398 | 49.00 |

*modified from https://github.com/joanam/scripts/blob/master/allelicBalance.py

**Table S5**. Summaries of reported analyses.

| **Analysis** | **Dataset** | **Linkage pruned** | **Number of SNPS** | **Program** |
| --- | --- | --- | --- | --- |
| Principal Component Analysis | Regional | Yes | 1409 | Plink 1.90b4.9 |
| MANOVA | Regional | Yes | 1409 | R 4.0.0 + heplots 1.3-5 |
| ADMIXTURE | Regional | Yes | 1409 | Plink 1.90b4.9 + ADMIXTURE 1.3.0 |
| fineRADstructure | Regional | No | 1997 | fineRADstructure 0.2 + fineSTRUCTURE 4.0.1 |
| Linear Discriminant Analysis | Regional | Yes | 1409 | MASS 7.3-51.6 |
| Observed heterozygosity | Regional | Yes | 1409 | VCFtools 0.1.16 |
| Nucleotide diversity | Regional | No | 1997 | VCFtools 0.1.16 |
| Tajima’s D | Regional | No | 1997 | VCFtools 0.1.16 |
| Private alleles | Regional | No | 1997 | Stacks 2.53 |
| Pairwise Weir and Cockerham’s Weighted Fst | Regional | No | 1997 | VCFtools 0.1.16 |
| Pairwise Absolute Divergence | Regional | No | 1997 | popgenWindows.py |
| Pairwise Euclidean Distances | Regional | Yes | 1409 | Plink 1.90b4.9 + R 4.0.0 |
| Isolation by Distance: Fst | Regional | No | 1997 | Raster 3.1-5 + R 4.0.0 + ade4 1.7.15 |
| Isolation by Distance: Dxy | Regional | No | 1997 | Raster 3.1-5 + R 4.0.0 + ade4 1.7.15 |
| Isolation by Distance: Euclidean Distances | Regional | Yes | 1409 | Raster 3.1-5 + R 4.0.0 + ade4 1.7.15 |
| Genome scans: Fst | Regional | No | 1997 | VCFtools 0.1.16 + R 4.0.0 |
| Genome scans: Dxy | Regional | No | 1997 | popgenWindows.py + R 4.0.0 |
| Genome scans: Tajima’s D | Regional | No | 1997 | VCFtools 0.1.16 + R 4.0.0 |
| Genome scans: Pi | Regional | No | 1997 | VCFtools 0.1.16 + R 4.0.0 |
| OutFLANK Fst outlier analysis | Regional | No | 1997 | OutFLANK 0.2 + R 4.0.5 |

**Figure S3**. Log likelihood for each *K* in Admixture (regional dataset).

**Figure S4**. fineRADstructure grouping of individuals from all populations from the regional dataset. While five groups of individuals had high support >0.07, including three with >0.8, these all consisted of individuals from geographically scattered populations, potentially carrying shared rare alleles. These three groups were Aneby (239) + Strömsund (288), Skruvemåla (166) + Nordmaling (206), Borås (186) + N6400 (23) + Karlskrona (269).

**Figure S5**. Pairwise Euclidean genetic distances between populations.

**Figure S6**. The relationship between geographical distance and pairwise genetic differentiation measured as Euclidean distances between populations (r=0.209, p=0.0499).

**Figure S7**. Outlier loci between identified for additional population genetic parameters. A) absolute divergence Dxy, between the northern and southern groups. B) Nucleotide diversity, pi. C) Selection, global Tajima’s D.

**Table S6**. Summary of outliers beyond the 99 % quantile.

| **Scaffold name** | **Middle coordinate in scaffold of coheerent region** | **Number of 100 kb windows forming region** | **Average number of SNPs per window** | **Statistic** | **Average statistic value per window** | **Annotation name** | **Description of Annotation** | **Location of middle coordinate relative to annotation** | **PFAM domains** | |
| --- | --- | --- | --- | --- | --- | --- | --- | --- | --- | --- |
| IpsContig2 | 15587501 | 4 | 1 | Dxy | 0.500 | Ityp02941 | uncharacterized protein LOC111692276 | 6.4 Kb Downstream | N/A |  |
| IpsContig3 | 1050001 | 1 | 20 | Pi | 3.23E-05 | Ityp05687 | adrenodoxin-like protein, mitochondrial | 1.9 Kb Upstream | Fer2 | PF00111 |
| IpsContig5 | 5850001 | 1 | 3 | Tajima's D | 2.31 | Ityp03628 | hypothetical protein YQE_09626, partial | 837 bp Upstream | 7tm_7 | PF08395 |
| IpsContig5 | 8312501 | 4 | 1 | Dxy | 0.528 | Ityp03981 | protein couch potato isoform X3 | 1.6 Kb Upstream | RRM_1 | PF00076 |
| IpsContig11 | 5950001 | 1 | 2 | Tajima's D | 2.36 | Ityp10355 | hypothetical protein B5V51_4051 | Within (Intronic) | RVT_1 | PF00078 |
| IpsContig14 | 825001 | 7 | 15 | Pi | 3.52E-05 | Ityp12430 | serine/threonine-protein kinase MARK2-like isoform X17 | Within (Exonic) | N/A |  |
| IpsContig14 | 2100001 | 3 | 12 | Pi | 2.98E-05 | Ityp12641 | protein MAATS1-like | Within (Exonic) | CFAP91 | PF1473 |
| IpsContig14 | 3637501 | 4 | 1 | Fst | 0.156 | Ityp12817 | tyrosine-protein kinase-like otk | Within (Exonic) | Pkinase_Tyr | PF07714 |
| IpsContig18 | 1750001 | 1 | 6 | Tajima's D | 4.09 | Ityp15175 | PREDICTED: uncharacterized protein LOC109546387 | 17.8 Kb Upstream | N/A |  |
| IpsContig18 | 1750001 | 1 | 6 | Tajima's D | 4.09 | Ityp15176 | calpain-A-like isoform X1 | 10 Kb Downstream | Calpain_III | PF01067 |
| IpsContig18 | 2337501 | 4 | 1 | Fst | 0.160 | Ityp15302 | PREDICTED: roquin-1 | Within (Exonic) | ROQ_II | PF18386 |
| IpsContig22 | 87501 | 4 | 1 | Dxy | 0.501 | Ityp14401 | hypothetical protein TcasGA2_TC032855 | Within (Intronic) | N/A |  |
| IpsContig84 | 287501 | 4 | 10 | Fst | 0.147 | Ityp21085 | Down syndrome cell adhesion molecule-like protein Dscam2 | Within (Exonic) | I-set | PF07679 |

**Table S7**. Summary of outliers from OutFLANK with a q-threshold lower than 0.01.

| **Scaffold name** | **Outlier coordinate in scaffold** | **Expected Heterozygosity** | **Corrected Fst** | **Uncorrected Fst** | **q-value** | **p-value** | **Annotation name** | **Description of Annotation** | **Location of coordinate relative to annotation** | **PFAM domains** | |
| --- | --- | --- | --- | --- | --- | --- | --- | --- | --- | --- | --- |
| IpsContig14 | 4910067 | 0.135 | 0.157 | 0.170 | 0.006 | 1.3E-04 | Ityp12858 | thyrotroph embryonic factor | Within (Intronic) | bZIP_2 | PF07716 |
| IpsContig14 | 4910068 | 0.135 | 0.157 | 0.170 | 0.006 | 1.3E-04 | Ityp12858 | thyrotroph embryonic factor | Within (Intronic) | bZIP_2 | PF07716 |
| IpsContig18 | 2336097 | 0.134 | 0.160 | 0.172 | 0.006 | 1.2E-04 | Ityp15302 | PREDICTED: roquin-1 | Within (Exonic) | ROQ_II | PF18386 |
| IpsContig84 | 278337 | 0.168 | 0.208 | 0.218 | 0.002 | 8.7E-06 | Ityp21084 | Down syndrome cell adhesion molecule-like protein Dscam2 | Within (Intronic) | I-set | PF07679 |
| IpsContig84 | 278402 | 0.152 | 0.183 | 0.194 | 0.004 | 3.3E-05 | Ityp21084 | Down syndrome cell adhesion molecule-like protein Dscam2 | Within (Intronic) | I-set | PF07679 |
| IpsContig84 | 282184 | 0.163 | 0.220 | 0.230 | 0.002 | 4.3E-06 | Ityp21084 | Down syndrome cell adhesion molecule-like protein Dscam2 | Within (Exonic) | I-set | PF07679 |
| IpsContig84 | 295779 | 0.191 | 0.161 | 0.172 | 0.006 | 1.1E-04 | Ityp21089 | tyramine beta-hydroxylase | Within (Exonic) | DOMON | PF03351 |
| IpsContig84 | 295797 | 0.191 | 0.161 | 0.172 | 0.006 | 1.1E-04 | Ityp21089 | tyramine beta-hydroxylase | Within (Exonic) | DOMON | PF03351 |
| IpsContig84 | 295848 | 0.191 | 0.161 | 0.172 | 0.006 | 1.1E-04 | Ityp21089 | tyramine beta-hydroxylase | Within (Exonic) | DOMON | PF03351 |
